# Tricuspid Valve Endocarditis due to *Streptococcus bovis* in a Patient with Ventricular Septal Defect: A Rare Manifestation—Case Report and Review of the Literature

**DOI:** 10.1155/2017/7152902

**Published:** 2017-11-01

**Authors:** J. Pushpakumara, G. Mudiyanse, A. Jayawardana, J. Siriwardana, N. L. A. Shyamali, J. Indrakumar

**Affiliations:** ^1^University Medical Unit, Colombo South Teaching Hospital, Kalubowila, Sri Lanka; ^2^Department of Medicine, Faculty of Medical Sciences, University of Sri Jayewardenepura, Nugegoda, Sri Lanka

## Abstract

*Streptococcus bovis* endocarditis has 18%–62% association with colonic neoplasms with multivalvular involvement and affects mainly elderly males leading to severe cardiac dysfunction, septic embolization, and neurological complications. The aortic valve is the commonest valve to be affected followed by aortic and mitral valves together. However, involvement of tricuspid valve is extremely rare. There are no reported cases of *Streptococcus bovis* endocarditis affecting the tricuspid valve in the presence of ventricular septal defect with left to right shunt. We report the case of a 25-year-old female with ventricular septal defect who was diagnosed to have tricuspid valve endocarditis caused by *Streptococcus bovis*. Her detailed colonoscopy, upper gastrointestinal endoscopy, liver biochemistry, and ultrasound scan of the abdomen were normal. She made a very good recovery with six weeks of intravenous antibiotics. This is the first case of tricuspid valve endocarditis caused by *Streptococcus bovis* in association with ventricular septal defect without any colonic lesions.

## 1. Introduction

*Streptococcus bovis* endocarditis is known to occur in patients with colonic carcinoma or adenoma and chronic liver disease [1–4]. The association of *S. bovis* bacteremia with colonic neoplasms ranges from 25% to 80% while endocarditis occurs only in 18%–62% [5]. The natural history of *S. bovis* endocarditis is the involvement of more than one valve, most frequently aortic and mitral valves together, leading to bad prognosis and frequent septic embolization [1, 3]. We report a young female with ventricular septal defect who was diagnosed to have tricuspid valve endocarditis due to *S. bovis* in the absence of any colonic neoplasms, upper gastrointestinal lesions, or chronic liver disease. Extensive literature review revealed no such case reported in the world literature.

## 2. Case Presentation

A 25-year-old female with uncomplicated ventricular septal defect presented to our casualty medical ward with the complaint of intermittent low-grade fever of 3-month duration. She had generalized malaise, loss of appetite, and weight loss over the same period. There was no history suggestive of chronic respiratory illnesses, tuberculosis, or connective tissue disorders. She was diagnosed to have small ventricular septal defect (VSD) since childhood and was on regular echocardiographic evaluation. She did not have history or peripheral evidence of intravenous drug abuse. On examination, she was thin built, pale, and febrile. There was no lymphadenopathy or features of autoimmune connective tissue disorders. She did not have finger clubbing. Her pulse rate was 90 beats per minute and blood pressure was 110/70 mmHg. There was no cardiomegaly. She had a left parasternal thrill and a harsh pan systolic murmur over the left lower sternal edge. There were no features of heart failure. Other systems' examination was unremarkable.

**Figure 1:**
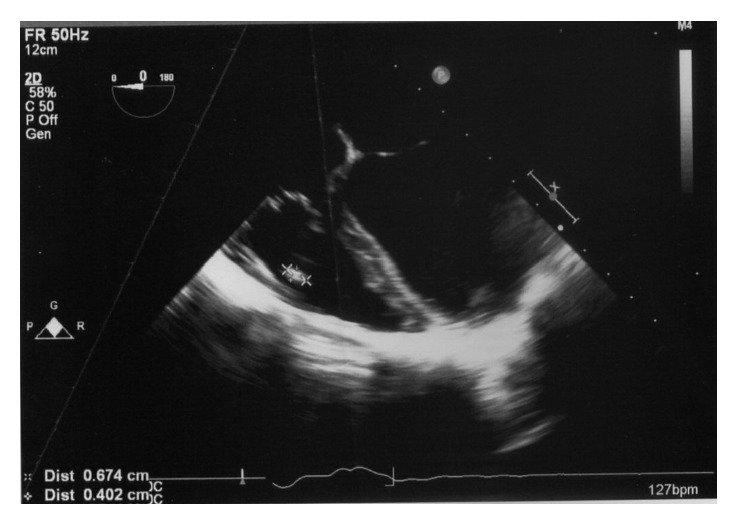
Vegetation (7 × 4 mm) seen on the tricuspid valve in transoesophageal echocardiogram (TOE).

**Figure 2:**
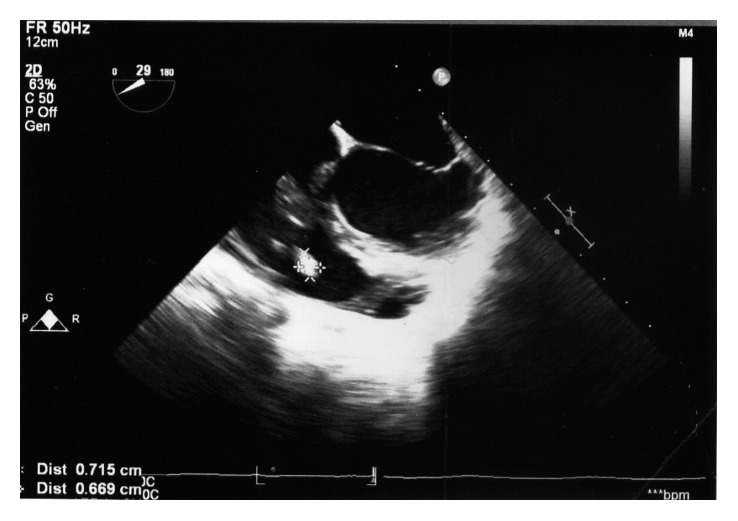
TOE after 2-week treatment: vegetation size remained the same.

Her investigations revealed white blood cell count 14 × 10^3^/µl, neutrophils 75%, lymphocytes 18%, hemoglobin 8.8 g/dl, platelets 224 × 10^3^/µl, erythrocyte sedimentation rate (ESR) 40 mm in 1st hour, C-reactive protein (CRP) 75 mg/l, alanine transaminase (ALT) 26 IU/l, and aspartate transaminase (AST) 30 IU/l. Anti-nuclear antibodies (ANA) were negative. The electrocardiogram (ECG) showed sinus tachycardia. Chest radiograph was normal. The 2D echocardiogram (2DE) revealed a perimembranous VSD, left to right shunt with a maximum pressure gradient of 105 mmHg. There was moderate tricuspid regurgitation with a maximum tricuspid pressure gradient of 36 mmHg. There was 7 mm × 4 mm-sized vegetation attached to the posterior leaflet of the tricuspid valve. The pulmonary valve was free of vegetations. There was moderate pulmonary hypertension. Her transoesophageal echocardiogram (TOE) confirmed the tricuspid valve vegetation (Figures 1 and 2). There were no abnormalities seen in other cardiac valves. Her blood culture was positive for *Streptococcus bovis* in two samples. She was diagnosed to have infective endocarditis based on fulfilling modified Duke's criteria and empirically started on intravenous ceftriaxone and gentamicin. Subsequent culture confirmed that the organism was sensitive to empirical antibiotics, hence continued. She also underwent detailed colonoscopy and upper gastrointestinal endoscopy, and both were normal. Her ultrasound scan of the abdomen did not reveal any abnormality. She was given intravenous gentamicin for 2 weeks and ceftriaxone for 6 weeks and made an uneventful recovery. The patient was referred to the cardiology department for further assessment for closure of VSD.

## 3. Discussion

Infective endocarditis is defined as the infection of the endocardial surface of the heart [6]. However, isolated tricuspid valve endocarditis (TVE) is uncommon and seen in 10%–15% of patients with infective endocarditis. *Staphylococcus aureus* is the commonest organism which causes TVE, especially in intravenous drug users (IDU) and patients with central venous catheters [7]. Tricuspid valve endocarditis due to *S. bovis* is rare, and the literature review revealed only a few cases. However, *S. bovis* endocarditis on the tricuspid valve in the presence of VSD has not been reported in the world literature.

*Streptococci bovis* (Gram-positive cocci) belongs to group D of the Lancefield classification and colonizes the gastrointestinal tract of the 2%–15% of the normal population [2, 4]. *Streptococcus bovis* endocarditis has been associated with colonic neoplasms and chronic liver diseases. However, the reasons underlying the association of colonic neoplasms and the *S. bovis* bacteremia are not known [8]. Recently published data suggest that *S. bovis* endocarditis has epidemiological differences depending on the geographical source [9, 10].

A case series published in 2015 demonstrated the major characteristics of *S. bovis* endocarditis [1]. These cases were reported from 2005 to 2014 in Latin America. There were a total of 9 patients of which 7 were males while 2 were females, and all of them fulfilled Duke's criteria for infective endocarditis. The key feature was that all the patients had had involvement of the native aortic valve. Furthermore, about 77.7% (7/9) patients had abnormal colonoscopic findings. *Streptococcus bovis* endocarditis was mostly seen in elderly males whose age was more than 50 years, which has been observed in several case series in France, Germany, Italy, and Ireland [1, 4, 11]. The most frequently affected valve was aortic followed by mitral and aortic together. This organism is responsible for about 13% of infective endocarditis and may affect more than one valve [2]. This results in severe valvular damage, hemodynamic instability, and frequent septic embolization leading to major neurological complications [3]. But our patient did not have colonic neoplasms or chronic liver disease as the predisposing factors for development of *S. bovis* endocarditis.

A patient with fever and multiple septic pulmonary embolization due to TVE has been reported in 2014 [12]. This patient, however, was initially thought to be a case of metastatic lung malignancies because of pulmonary symptoms. In fact, when there are multiple lung abscesses in non-IDUs, the possibility of *S. bovis* endocarditis of the tricuspid valve has to be suspected as a differential diagnosis.

An extremely severe case of TVE caused by *S. bovis* has been reported in 1996 [7]. This was a young male who had hepatitis B, presented with respiratory symptoms and fever due to septic pulmonary embolization caused by TVE. The initial transoesophageal echocardiogram (TOE) revealed vegetation on the septal leaflet of the tricuspid valve with significant regurgitation which was complicated with a new vegetation on the anterior leaflet after 2 weeks of hospital admission. The patient had become severely sick and required surgical excision of the tricuspid valve due to failed antibiotic therapy. However, the patient had recovered with prolonged intensive care. Subsequently, he had undergone a colonoscopic examination which was normal. In contrast, our patient presented with pyrexia of unknown origin (PUO) for 3 months. She did not have septic embolization and well responded to the antibiotics within the first week.

Beeching et al. have described the importance of rigorous investigation to exclude both endocarditis and neoplasms in the large bowel, in patients with bacteremia caused by *S. bovis* [8]. In this study, there had been 12 patients with *S. bovis* bacteremia from 1979 to 1984. There were 10 patients with endocarditis of which only one patient had TVE caused by *S. bovis*. This patient was an elderly male who presented with fever and constitutional symptoms shortly after resection of recto-sigmoid adenocarcinoma. Interestingly, this patient had a purpuric skin rash, painful arthritis of lower limbs, splenomegaly, and Roth spots in the right fundus. There was no evidence of intracardiac shunts such as VSD. The patient had developed severe vasculitis with reduced complement levels. In our patient, there were no such immunological phenomena observed.

These cases demonstrate the different clinical presentations of TVE caused by *S. bovis* which can give rise to a diagnostic dilemma. Our patient also had a preexisting pansystolic murmur due to congenital VSD which mimicked the murmur produced by tricuspid regurgitation caused by TVE. In addition, she did not have striking features of endocarditis other than the fever. Hence, a very careful examination and the clinical suspicion of endocarditis were extremely important.

Kupferwasser et al. had studied the clinical and morphological features of *S. bovis* endocarditis in 1998 [11]. A total of 177 patients who were diagnosed to have infective endocarditis based on Duke's criteria have been evaluated. There had been 22 cases of *S. bovis* endocarditis. The results showed *S. bovis* endocarditis occurred mainly in elderly people with involvement of multiple valves leading to severe hemodynamic instability. Moreover, *S. bovis* endocarditis had caused a higher mortality rate requiring cardiac surgical interventions as compared to endocarditis caused by other organisms such as other *Streptococci* and *Staphylococci*. However, in our patient, the age of onset was young and only the tricuspid valve affected. She did not have hemodynamic instability, hence recovered with antibiotics. This case illustrates the uncommon presentation of TVE (in the presence of VSD) due to *S. bovis*.

## 4. Conclusion

Unlike the other reported cases, our patient had unique features, including being a younger patient, presence of VSD, and the absence of immunological phenomena. Furthermore, our patient did not have other features to suspect *S. bovis*-infective endocarditis such as colonic neoplasms or chronic liver diseases.

This case illustrates an uncommon presentation of *S. bovis* endocarditis on the right heart which was due to left to right shunt via VSD. There were no published data on TVE due to *Streptococcus bovis* with coexistent VSD.

## References

[1] Melloa R., da Silva Santosb M., Golebiosvkib W., Wekslerb C., Lamasa C. (2015). *Streptococcus bovis* endocarditis: analysis of cases between 2005 and 2014. *Brazilian Journal of Infectious Diseases*.

[2] Ahamed Riyaaz A. A., Samarasinghe R., Sellahewa K., Sivakumaran S., Tampoe M. S. (2016). Native valve *Streptococcus bovis* endocarditis and refractory transfusion dependent iron deficiency anaemia associated with concomitant carcinoma of the colon: a case report and review of the literature. *Case Reports in Infectious Diseases*.

[3] Duval X., Papastamopoulos V., Longuet P. (2001). Definite *Streptococcus bovis* endocarditis: characteristics in 20 patients. *Clinical Microbiology and Infection*.

[4] Tripodi M. F., Adinolfi L. E., Ragone E. (2004). *Streptococcus bovis* endocarditis and its association with chronic liver disease: an underestimated risk factor. *Clinical Infectious Diseases*.

[5] Abdulamir A. S., Hafidh R. R., Bakar F. A. (2011). The association of *Streptococcus bovis/gallolyticus* with colorectal tumors: the nature and the underlying mechanisms of its etiological role. *Journal of Experimental & Clinical Cancer Research*.

[6] Turhan Ö., Saba R., Belgi A., Inan D., Karaoglan H., Yalcin A. N. (2005). A case of right-side infective endocarditis with ventricular septal defect. *Le Infezioni in Medicina*.

[7] Model A., Craig C. P. (1996). Isolated tricuspid valve endocarditis due to *Streptococcus bovis*. *Clinical Infectious Disease*.

[8] Beeching N. J., Christmas T. I., Ellis-Pegler R. B., Nicholson G. I. (1985). *Streptococcus bovis* bacteremia requires rigorous exclusion of colonic neoplasia and endocarditis. *Quarterly Journal of Medicine*.

[9] Olmos C., Vilacosta I., Sarria C (2016). *Streptococcus bovis* endocarditis: update from a multicenter registry. *American Heart Journal*.

[10] Corredoira J., Garcia-Pais M. J., Rabunal R., Alonso M. P. (2016). *Streptococcus bovis* endocarditis: epidemiological differences depending on geographical source. *American Heart Journal*.

[11] Kupferwasser I., Darius H., Müller A. M. (1998). Clinical and morphological characteristics in *Streptococcus bovis* endocarditis: a comparison with other causative microorganisms in 177 cases. *Heart*.

[12] Patricio I. M., Caetano F., Queiro J. C., Marado D. (2014). Keeping track of migratory pulmonary lesions. *BMJ Case Reports*.

